# Critical race theory as a tool for understanding poor engagement along the HIV care continuum among African American/Black and Hispanic persons living with HIV in the United States: a qualitative exploration

**DOI:** 10.1186/s12939-017-0549-3

**Published:** 2017-03-24

**Authors:** Robert Freeman, Marya Viorst Gwadz, Elizabeth Silverman, Alexandra Kutnick, Noelle R. Leonard, Amanda S. Ritchie, Jennifer Reed, Belkis Y. Martinez

**Affiliations:** 1 Brooklyn, NY USA; 2 Center for Drug Use and HIV Research (CDUHR), Rory Meyers College of Nursing, New York University, 433 First Avenue, New York, NY 10010 USA

**Keywords:** Qualitative, Critical race theory, HIV care continuum, African American, Black, Hispanic, Structural barriers, HIV/AIDS, Health equity, Antiretroviral therapy initiation

## Abstract

**Background:**

African American/Black and Hispanic persons living with HIV (AABH-PLWH) in the U.S. evidence insufficient engagement in HIV care and low uptake of HIV antiretroviral therapy, leading to suboptimal clinical outcomes. The present qualitative study used critical race theory, and incorporated intersectionality theory, to understand AABH-PLWH’s perspectives on the mechanisms by which structural racism; that is, the macro-level systems that reinforce inequities among racial/ethnic groups, influence health decisions and behaviors.

**Methods:**

Participants were adult AABH-PLWH in New York City who were not taking antiretroviral therapy nor well engaged in HIV care (*N* = 37). Participants were purposively sampled for maximum variation from a larger study, and engaged in semi-structured in-depth interviews that were audio-recorded and professionally transcribed verbatim. Data were analyzed using a systematic content analysis approach.

**Results:**

We found AABH-PLWH experienced HIV care and medication decisions through a historical and cultural lens incorporating knowledge of past and present structural racism. This contextual knowledge included awareness of past maltreatment of people of color in medical research. Further, these understandings were linked to the history of HIV antiretroviral therapy itself, including awareness of the first HIV antiretroviral regimen; namely, AZT (zidovudine) mono-therapy, which was initially prescribed in unacceptably high doses, causing serious side effects, but with only modest efficacy. In this historical/cultural context, aspects of structural racism negatively influenced health care decisions and behavior in four main ways: 1) via the extent to which healthcare settings were experienced as overly institutionalized and, therefore, dehumanizing; 2) distrust of medical institutions and healthcare providers, which led AABH-PLWH to feel pressured to take HIV antiretroviral therapy when it was offered; 3) perceptions that patients are excluded from the health decision-making process; and 4) an over-emphasis on antiretroviral therapy compared to other non-HIV related priorities. We found that although participants were located at the intersection of multiple social categories (e.g., gender, social class, AABH race/ethnicity), race/ethnicity and social class were described as primary factors.

**Conclusions:**

Critical race theory proved useful in uncovering how macro-level structural racism affects individual-level health decisions and behaviors. HIV clinical settings can counter-balance the effects of structural racism by building “structural competency,” and interventions fostering core self-determination needs including autonomy may prove culturally appropriate and beneficial for AABH-PLWH.

## Background

Treatments for HIV infection have improved dramatically in recent years [1]. These advances, in turn, have allowed persons living with HIV (PLWH) to experience a higher quality of life and a longer lifespan than in the earlier phases of the HIV pandemic [2, 3]. Despite these improvements, however, serious gaps in the HIV care continuum persist in the United States; namely, insufficient rates of initial diagnosis, linkage to care, retention in care, prescription and uptake of antiretroviral therapy (ART), and viral suppression [4]. For example, recent data from the Centers for Disease Control and Prevention estimate of the 1.2 million PLWH in the United States, only 37% are prescribed ART, and less than a third (30%) are virally suppressed [4, 5]. Further, racial/ethnic disparities in HIV health outcomes are marked, with African-American/Black and Hispanic PLWH (AABH-PLWH), who are primarily from lower socioeconomic status backgrounds, evidencing higher rates of morbidity and earlier mortality than their White peers [6], underscoring the structural underpinnings of the HIV epidemic [7]. Indeed, a substantial body of literature highlights that, compared to their White peers in the United States, African American/Black and Hispanic patients have worse access to and more negative experiences of health care settings, as well as inferior health outcomes [8–10], including among PLWH [11, 12], highlighting the importance of eliciting the perspectives of AABH-PLWH, in order to understand and better address racial/ethnic disparities in HIV.

The literature highlights a number of factors that contribute to AABH-PLWH engaging in HIV care at suboptimal levels and/or to delay, decline, or discontinue ART. This literature to date has focused primarily on barriers at the individual level of influence, such as unsupportive health beliefs, including negative outcome expectancies regarding ART, low self-efficacy to manage ART, and negative emotions, including fear [13, 14], as well as substance use [15], and mental health concerns, primarily depression [16, 17]. Further, lack of knowledge about HIV care/ART guidelines may impede care and ART uptake [18], and AABH-PLWH often decline ART because they believe they lack skills to adhere to ART regimens [19, 20]. Some research has focused on barriers at the social level of influence and found a lack of peer role models who are regularly engaged in HIV care and adhering to ART, social isolation, and low levels of social support impede engagement along the HIV care continuum [21–23]. Further, fear of HIV stigma, compounded by the stigma associated with poverty, substance use, and/or sexual minority status, also serve as barriers to engagement along the HIV care continuum for AABH-PLWH [24, 25]. Theoretically, these barriers work together synergistically to reduce motivation to engage in HIV care and/or initiate ART.

Structural barriers are aspects of the larger external environment that limit individuals’ options. These include general social and health-specific policies (e.g., related to criminal justice practices and residential segregation), aspects of organizations (e.g., availability of services, ease of access), and/or infrastructure (e.g., lack of transportation available to high-quality health care settings) [26]. Past research has identified environmental and organizational structural factors that impede optimal engagement along the HIV care continuum for AABH-PLWH, including difficulties they may experience negotiating the health care system, problems with insurance [27], and financial and transportation challenges [28]. However, in comparison to the literature on individual- and even social-level barriers, substantially less is known about structural barriers and how these are experienced by AABH-PLWH in relation to HIV care and ART. Indeed, the World Health Organization has called for the study of such social/structural determinants of health and their effects on resources to promote health [29].

To address this gap in the literature, we conducted a qualitative study informed by critical race theory (CRT), and incorporating intersectionality – complementary trans-disciplinary theoretical frameworks grounded in social justice [30–32]. CRT is designed to illuminate contemporary racial phenomena, expand the discourse about complex racial concepts, and challenge racial hierarchies. As a research methodology, CRT elicits “counter-narratives” that take the lived experiences of people of color at face value in order to illuminate the ways they experience living in an unequal society; that is, one characterized by structural racism [31]. Structural racism is defined as the macro-level systems that create, sustain, and reinforce inequities among racial and ethnic groups [33]. The forms and effects of structural racism include social segregation, disproportionate criminalization, and unequal resources, including inequalities in access to high-quality, personalized HIV care, all of which are overlapping and mutually reinforcing.

CRT proceeds from an understanding that while structural racism is less visible than individual racism, it is just as, if not more, influential. Unlike individual racism, structural racism is a systemic, historically rooted form of oppression that cannot be eradicated simply at the level of individual attitudes or behavior. Indeed, the individuals operating within institutions may be, in practice, non-discriminatory, but still operate within a larger structurally racist context. For example, a health care provider may understand him or herself as delivering high-quality care to all his/her patients regardless of their race and backgrounds, but this provider nonetheless operates within an institutional context marked by unequal resources, policies, and practices. Yet as a result of failing to address the ways in which health care providers in particular, and the medical system in general, are perceived by patients who have experienced multiple, overlapping forms of discrimination, many health care providers may nonetheless inadvertently contribute to AABH-PLWH’s experiences of discrimination within health care settings.

Through eliciting counter-narratives, CRT guides an exploration of how institutional policies and structures, as well as prevailing cultural norms, work to influence the health care decisions and outcomes among populations of color [34]. The aim of the present qualitative study is to use CRT to elucidate the mechanisms by which structural racism might shape and influence AABH-PLWH’s relationships with HIV care settings and health decisions, particularly within the context of HIV care and ART uptake, while also attending to intersections of other frequently marginalized social categories. Thus, in addition to CRT, we drew on the theory of intersectionality to understand how the combination of disadvantaged positions plays out in health care settings [31]. In keeping with the larger literature on the intersectional nature of social categories such as race/ethnicity, gender, HIV status, and social class [32, 35, 36], we attended where possible to the ways these intersections might create overlapping and interdependent systems of discrimination or disadvantage.

### The local context

The present study was conducted in New York City (NYC), a location with a large and mature HIV epidemic comprised of approximately 120,000 PLWH, more than 75% of whom are African-American/Black and Hispanic [37]. NYC has achieved higher rates of engagement along the HIV care continuum compared to national figures (e.g., 43% in NYC are virally suppressed [38] compared to 30% nationally [5]). The city hosts a large network of HIV care facilities and related support services for those insured and uninsured [39] and all patients have access to ART at low or no cost [40]. Nonetheless, NYC evidences serious gaps in engagement along the HIV care continuum. First, many PLWH are diagnosed late in the course of their HIV disease [41]. Racial/ethnic disparities in engagement in care, ART initiation, and viral suppression are similar to national patterns, where AABH-PLWH show the lowest rates of engagement along the continuum [42]. Further, HIV prevalence and HIV-related death rates are highly concentrated in the highest-poverty neighborhoods of NYC, which are predominantly African-American/Black and Hispanic, highlighting the structural underpinnings of racial/ethnic disparities in HIV/AIDS [43].

## Methods

### Recruitment

Participants were selected from a larger study of AABH-PLWH (*N* = 95) to develop interventions to increase ART initiation among those who were medically eligible for ART, but had delayed, declined, or discontinued ART, many of whom were also poorly engaged in HIV primary care. Participants for the larger study were recruited mainly through peer-to-peer referrals in New York City in 2012–2014. For the present qualitative study, participants were purposively sampled from this larger cohort for maximum variation on HIV history (e.g., diagnosed within the past 5 years and more than 5 years ago) and ART experiences (e.g., never took ART in the past, took ART at least once in the past, stopped and started ART frequently in the past). A total of 37 participants were sampled, approached, and enrolled in the qualitative study, for which participants gave signed informed consent. None of those approached declined to participate. Interviews took place 4–6 months after enrollment in the larger study. Procedures were approved by the Institutional Review Boards at New York University and two collaborating hospital sites. The larger study is described in more detail elsewhere [17, 44].

### Procedures

Participants selected for the present study were contacted by study staff via telephone to schedule one-on-one, in-person interviews at a community-based field site. Interviews lasted 60–90 min and were conducted by research staff members trained in qualitative methods. Interviews followed a semi-structured interview guide to explore participants’ personal histories of HIV care and ART initiation, as well as their perspectives on individual, social, and structural factors that may influence engagement in HIV care and decisions about ART initiation and continuation, informed by the theories and literature reviewed above. The semi-structured format included main questions and probes, and allowed for the emergence of themes not identified *a priori*. Interviews were conducted until data saturation was reached on core constructs [45, 46], as determined by a senior qualitative researcher, who reviewed transcripts and queried interviewers during data collection. Interviews were audio-recorded and professionally transcribed verbatim for analysis.

### Analysis

We used a systematic content analysis approach [47, 48] that was both theory-driven and inductive. Data were analyzed in the Dedoose platform. First, the research team developed a set of initial descriptive codes based on a review of transcripts, reflecting the domains of the research question and theoretical models (e.g., stopping ART, feelings about ART, settings where HIV care is provided, relationships with providers, emotions, race/ethnicity, and gender). The analysis took place within an “interpretive community” [49, 50]; that is, a small group of four multidisciplinary scholars who independently read and coded interview excerpts. A trained primary qualitative analyst coded each interview transcript using these codes, refining and creating additional codes when necessary. At regular intervals through the coding process, a reliability check of the coding was conducted with members of the interpretive community, two of whom coded all transcripts, and one who coded a sub-set (25%). In this manner we established inter-coder reliability and further refined the existing set of codes. Discrepancies in coding were resolved by consensus. Codes relevant to CRT, such as race, ethnicity, class, institutions, structures, organizations, discrimination, historical factors, distrust, stigma, specific counter-narratives, experimentation, and pressure, were selected for further analysis. We also attended to the intersectional nature of social categories such as race/ethnicity, gender, HIV status, and social class [32]. Then, we used the Memo and Code Co-occurrence functions within Dedoose to support the analysis of relationships between and among codes and to thereby develop larger themes. Next, the interpretive community together identified areas of congruence and discrepancy with respect to the broader themes. The final analysis focused on those themes most closely aligned with the tenets of CRT and intersectional theory, exploring both manifest and latent constructs [51]. Methodological rigor of the analysis was maintained through an audit trail of process and analytic memos and periodic debriefing with the larger research team, which included experts in HIV care continuum issues, CRT, and intersectionality [52]. (Note we did not use the term “conspiracy theory” in the analysis. Such terms are inconsistent with the CRT approach, as they imply the understandings inherent in such counter-narrative explanations are or may be invalid. Instead, CRT underscores the validity and relevance of counter-narratives in order to take in the perspectives of populations “on the margins.”).

## Results

### Participant characteristics

As shown in Table 1, participants ranged in age from 26 to 64 years (*M* = 48.7 years, SD = 9.4 years). Most (59.5%) were male, and among the male participants, 59.1% identified as gay, bisexual, or otherwise non-heterosexual. Most participants (78.4%) were African American/Black and 21.6% were Hispanic. Virtually all (97.3%) were from lower socio-economic status backgrounds. Most (78.5%) had run out of funds for necessities, such as food, in the past year. Approximately two-thirds (64.9%) had been homeless over their lifetimes, but less than a quarter currently had unstable housing (18.9%). About half (56.8%) had a high school degree or higher. On average, participants had been diagnosed with HIV 13.9 years ago (SD = 8.1 years). Approximately half (54.1%) had taken ART at least once in the past, and on average, had initiated ART on 5.2 different occasions (SD = 5.4 times) prior to study enrollment. None were on ART at the time they enrolled in the study. Most (73.0%) rated their health as “good” or better, and the majority (83.8%) had an established health care provider in the past year, mainly at a hospital-based HIV clinic (77.8%). CD4 counts were relatively low (average CD4 = 291.9 cells/mm^3^ [SD = 144.2 cells/mm^3^]) and the mean HIV viral load level was substantial (mean log_10_ HIV viral load 3.1 [SD = 1.4]). All reported that a health care provider had recommended ART in the past. Half (50.0%) had symptoms of depression at a clinically significant level. Thus participants were diverse with respect to socio-demographic, health, and other background characteristics, in keeping with the sampling approach. A description of the psychometric characteristics of structured measures used to assess the sample are described elsewhere [44].

**Table 1:** Description of the sample (*N* = 37)

	Mean (SD) or %
*Socio-demographic characteristics*	
Age in years	48.69 (9.37)
Male sex	59.46
If male, gay, bisexual, non-heterosexual identity	59.10
African-American/Black, Not Hispanic	78.38
Latino/Hispanic	21.62
Low socioeconomic status	97.30
*Other background characteristics*	
Ran out of funds for basic necessities in past year	78.48
Ever homeless	64.92
Current housing instability	18.91
High-school degree or higher	56.81
*Health indicators*	
Health self-rating “good” or better	72.97
Receives Medicaid (public health insurance for low-income populations)	62.23
Years since HIV diagnosis	13.88 (8.12)
Health care provider recommended ART (lifetime)	100.00
Has taken ART in the past	54.05
Number of times started/stopped ART	5.17 (5.44)
Had an established HIV care provider over the past year (from the Medical Record)	83.78
Receives care in hospital-based HIV clinic	77.78
Average CD4 in the year before Baseline (Medical Record)	291.88 (144.15)
Average log_10_ viral load in the year before Baseline (Medical Record)	3.08 (1.36)
Depression screener at a clinically significant level	50.00

### Overview of results

Drawing on the experiential knowledge of AABH-PLWH, we found four primary themes describing how aspects of structural racism such as social segregation, concentrated poverty, and unequal resources informed experiences with and attitudes towards healthcare institutions – attitudes that, in turn, shaped individuals’ decisions whether or not to initiate ART and/or to engage in HIV care at recommended levels. We found participants’ social positions, with regard to race/ethnicity, along with class, and, to some extent, gender, worked together to create and reinforce what they experienced as a subordinate position in relation to the health care providers. That is, despite the importance of social class and gender in these analyses, participants explicitly identified race/ethnicity as the primary factor negatively affecting their experiences in the healthcare system, with social class as an important secondary influence. We did not find that racism or discrimination related to race/ethnicity was necessarily more important than other forms of marginalization, but instead, a more visible aspect of how participants viewed themselves within the context of structural racism. We found AABH-PLWH’s HIV care and ART decisions were experienced through a number of historical and cultural lenses which foregrounded past abuses of populations of color in medical research, such as the Tuskegee Syphilis Study [53, 54]. Partly as a result, AABH-PLWH were likely to view invitations to initiate ART with suspicion. With respect to specific themes, as we describe in detail below, we found structural racism as defined above contributed to 1) perceptions of healthcare settings as overly institutionalized, 2) distrust of medical institutions and healthcare providers, 3) the perception that patients are excluded from the health decision-making process, and 4) an overemphasis on ART over and above other non-HIV related priorities, all of which, in turn, created a sense of dehumanization. Results indicated these experiences of healthcare settings and the healthcare industry were highly interrelated, and together contributed to individuals’ finding healthcare settings unreceptive or even hostile, and, in many cases, antithetical to their healthcare needs. Yet we also found, as might be expected, participants expressed their desires to achieve or maintain good health, and despite strains experienced at the structural or organizational levels, many had productive relationships with individual health care providers. We wish to note not all statements made by participants would be considered factually accurate from the perspectives of the medical establishment. Instead, consistent with the CRT approach, they represent the perspectives of AABW-PLWH who have barriers to HIV care and ART, and are taken at face value. Names presented below are pseudonyms and identifying details have been changed to protect confidentiality.

#### The experience of “institutionalized” healthcare settings

We found participants in the present study engaged with a wide range of health care, social service (for government entitlements and housing support), community-based, and in some cases, criminal justice settings, located in areas with both higher and lower surrounding poverty rates. The extent to which participants experienced clinical settings as “institutionalized;” that is, closely resembling these other highly structured institutions through which they, as people from the lower socioeconomic strata, frequently travelled, was closely related to the degree to which they experienced distrust of and a lack of motivation to engage with their health care providers. Participants routinely described these more institutionalized clinical care settings as creating a sense of disempowerment, which was then linked to the degree they felt pressured by providers to initiate ART, and this, in turn, fostered a desire to avoid these settings. Relatedly, perceptions of racial/ethnic and economic disparities in the quality of HIV care were a common theme. Health care settings located in predominantly African American/Black and Hispanic neighborhoods were compared negatively to those in higher-income and mostly White neighborhoods. Noting the similarities between hospitals and clinics located in lower-income neighborhoods and other bureaucratic institutions, participants described being stereotyped and “*feeling like a number*” when seeking HIV care. Leon, a 47-year-old African American man who was not taking ART nor well engaged in HIV care, compared the healthcare facility where he sporadically received HIV care to a scaled down version of a notorious local jail complex—“*a baby Riker’s Island*”—and described his experience as follows:

*It really was more about the (medical) setting. I didn’t really feel comfortable by it. I think that the setting made me feel uncomfortable. Felt like the furniture and, you know, I felt like reception and people that worked there were just like paper pushers, and I didn’t really feel comfortable.... I felt like just something invisible was speaking to me. Just that the furniture was run down, the receptionist just seems caught up in a sense like people needing a vacation…I felt that the space wasn’t comfortable enough for me to want to sit there, and register. Just even if I was interested in going there, which I wasn’t, the atmosphere had made me not want to.*

Leon’s description of public health care facilities as leading one to feel “*lost in the crowd*” was typical in highlighting not only the impersonal nature of the facility’s staff, but of the physical setting itself. These impressions, and their negative associations with other organizations and structures, including the criminal justice system, played a crucial role in many participants’ overall experiences with HIV care.

Further, we found participants reported they were likely to receive lower quality care based on their geographical location and its presumed relationship with race/ethnicity and social class background, especially related to the amount of time that providers spent with them. Marcus, a 46-year-old African American man diagnosed with HIV over six years ago, described his experiences with HIV care institutions as degrading and demoralizing. He understood the dehumanizing and anonymizing way he was treated as based on both his actual and presumed socio-demographic characteristics, all linked to where he lived, a borough in New York City:

*I live in Brooklyn, and it’s funny because when you go the clinic or if you go even to, let’s say, the SSI office, (the) hospital, any of those major hospitals that have major clinics in them, you tend to be treated like a number and not a person. And some would say, oh, it’s discriminative, but it’s a little bit deeper than that. It’s the politics of the demographic of where you live, basically. This is what goes on in this neighborhood, so you get characterized as this and put in a box. Which isn’t always cool…I think basically it really has a big deal to do with where you live at. And then [health care providers] have all these other assumptions of the drugs, the crime. All these different things, and it’s just a different form to me of racial profiling…Depending on what street you live on makes a big difference between how you’re going to be treated. Soon as [they] find out your address-it changes a lot of things.*

We found healthcare settings were described as emitting “invisible” hostility that resulted in AABH-PLWH being denied individualized attention and treatment. This was exacerbated when participants felt they were perceived as less deserving of quality HIV care on the basis of overt racial/ethnic and class profiling. Darryl, a 60-year-old African American man diagnosed 17 years ago, who had never taken ART, summed up his past health care experiences:

*I think mostly it’s pains that mostly people of color go through. You know it’s a lot of pains. It’s a lot of rejection. Not understanding the person’s, you know, history or what that person’s been through, and trying to find someone that really cares to listen to that person. You don’t get much of that because they tell you, “I don’t have time because I have 50 other thousand people that have worse situations than you,” and so you walk out bitter. … So a lot of people—okay, a lot of African Americans —they are on that pre-judgment.*

Thus while not all participants reported experiences of overt racial/ethnic and/or class-based discrimination on the part of individual staff in health care settings, they nonetheless pointed out “deeper” and more “invisible” experiences of being treated unfairly and as a member of multiple, mutually reinforcing dehumanized stereotyped categories – inequitable aspects of the larger health care environment that could be described as consistent with structural racism.

#### The structural correlates of medical distrust

We found the theme of medical distrust was pervasive and powerful, and this distrust had profound effects on engagement in HIV care and uptake of ART. Distrust manifested variously among participants as suspicions they would be “guinea pigs” subject to medical experimentation, or concerns that decisions made by individual healthcare providers were overly influenced by the financial motivations of pharmaceutical companies.

Further, distrust was linked to fears that the side effects patients often experience when taking ART – more common in the earlier formulations of the medications – are evidence of its toxicity, and to some, even a form of genocide. For many participants, this specific aspect of distrust of ART is directly linked to the memory, or historical memory, of AZT monotherapy, one of the earliest treatments for HIV prescribed in the late 1980’s, which, as noted above, was prescribed in high doses, leading to serious side effects, but with only limited efficacy. In fact, AZT was commonly cited as the *cause* of death of many PLWH in the early days of HIV treatment. This “legacy” of AZT has been passed down among AABH-PLWH through the decades, indicating, to some PLWH, at least, malicious intent on the part of the pharmaceutical industry, and serving as a warning about the newer antiretroviral medications.

Despite great medical advancements in the treatment of HIV and a significant reduction in side effects experienced by patients, we found a legacy of medical experimentation, non-consensual treatment, and the maltreatment of people of color in healthcare persists, and specific counter-narratives continue to circulate and remain important sources of information guiding individuals’ healthcare decisions. The side effects of HIV medications, in general, remained some of the most easily articulated manifestations of medical distrust among AABH-PLWH within the overall historical context of the memory of past medical exploitation of populations of color.

Thus we found that a side effect was not always just a side effect. Indeed, for AABH-PLWH, ART side effects were experienced through a specific historical and cultural lens, often serving as a reminder of the individual’s location in an inequitable society. Indeed, we found ART side effects, or even just the fear of potential side effects, commonly produced major emotional barriers to ART initiation, continuation, and adherence.

Participants expressed a sense of vulnerability in relation to institutions they perceived as lacking concern for their well-being, or that, in some cases, which they felt sought to harm them. For some, this distrust was described as stemming from the historically documented medical exploitation of poor communities of color, especially poor African American/Black communities. For others, distrust of medical institutions was the result of an indefinable sense of wariness passed from generation to generation. Ramona is a 53-year-old African-American/Black woman who had frequently stopped and started HIV medications over the past 15 years since she was first diagnosed. She related a common, albeit highly disheartening, counter-narrative about an HIV/AIDS epidemic created and perpetuated by the government:

*I believe that they do have a cure out there but it’s gonna be for those that are very rich. Like, I’m gonna tell you, I believe the government made this. And they made it for the less desirables. And they didn’t expect their own kids to wind up (getting) it. And it spreaded. I’m not trying to be prejudiced or anything but they made it for the Blacks, the Puerto Ricans and they made it for the Whites that they didn’t want there.*

For Ramona, distrust of HIV treatment and the notion of a cure being withheld from the community of “less desirables” were directly related to her personal decisions to interrupt ART and HIV care. Moreover, lack of knowledge and suspected malicious intent on the part of healthcare providers and medical institutions about the efficaciousness and potential dangers of ART contributed to her understanding of taking ART as a type of experimentation, as she noted below:

*So what they basically doing is experimenting. They don’t have it correct yet. That’s why they’ve made so many different types. Sometime it make me feel that it’s not really worth taking.*

Victoria, an African American woman in her 40’s who had never sought medical care for HIV, expressed similar concerns:

*They like to experiment and say, okay, I heard of, what you call them, clinical trial, that’s what clinical trials is, one big guinea pig. That’s for all those guinea pigs. I know I’m a guinea pig, everybody’s a guinea pig.*

Julio, a 50-year-old Puerto Rican man who had frequently interrupted ART due to concerns about side effects, referred to the history of AZT as an example of how gaps in medical knowledge shape social norms of HIV medications among AABH-PLWH in particular:

*I would say my population of people are against medicines because they hear the old AZT, you know, you get fat belly. And you get more sicker. That was my idea at first too you know. Yes, a lot of current information isn’t being put out there so they’re still living on the old regimen medicines, AZT and all these other medicines that really, really got you really sick because they was just, I guess, learning. So a lot of my peers that don’t wanna take the medicines, [it’s] because they don’t want the added side effects of it.*

Moreover, Julio spoke extensively about the lack of trust he and his peers have in the information and financial motivations of medical institutions and pharmaceutical companies, which he believed have a disproportionate influence on already overburdened healthcare providers in economically depressed neighborhoods:

*I think it’s [social] class. I think some providers feel, they look down at the person. Not all, not all. I’ve had good providers, bad ones, but I feel like they [think they’re] better than [you], you know. “You come here for services from me? You know, who put you in that situation? You did.” You know what I’m saying? “Now you’re wasting my tax dollar,” you know what I’m saying? I can give you a perfect example. “No, I’m not giving you that med that’s too expensive.” Why not? Medicaid’s (government-provided health insurance for low-income patients) paying for it or whatever…but the doctor feels he don’t want to give him that medicine because they’re in bed with the pharmaceutical companies because the provider also get a little incentive. [The pharmaceutical companies say], “Here’s a vacation doc. Here’s a couple hundred dollars, you know, just promote this.” And I, me with my own eyes seen it, like that, the medicine for the belly. I’m in my clinic with my doctor and here comes the representative of this medicine, coming in trying to push it on me.*

We found participants were acutely aware of how structural racism and economic inequality take not only the form of outright discrimination or unintended micro-aggressions but, more importantly, operate as over-arching and invisible features of the healthcare industry. Yet in this context, and despite fear and distrust, we found participants were continually evaluating their health care needs, seeking services, trying medications (including ART in some cases), and engaging in health care. We found participants were emotionally torn between, or perhaps immobilized by, the desire to engage in health care and take ART, and their strong desires to avoid negative reactions received from, and elicited by, health care settings and encounters. Thus medical distrust often impeded, but did not necessarily preclude, involvement in health care.

#### The importance of autonomy in medical decision-making

We found the need among participants to make confident, informed decisions about initiating ART “*when it is right for (them)*” was another common and potent theme. This desire for autonomy was often discussed in opposition to the experience of being pressured and even judged by health care providers, who, participants noted, pressed them to immediately begin ART after their initial HIV diagnosis, regardless of other life factors such as treatment anxiety, lack of emotional readiness, fear of stigma, substance use problems, or other health conditions. Moreover, the perceived privileging of medical expertise over the patient’s own knowledge, experience, and immediate concerns caused patients to feel excluded from the ART decision-making process, thereby undermining their sense of self-determination with respect to their health and their body. This contributed to a feeling that providers often doubted whether patients had the capacity to make decisions in their own best interest.

Marcus (described above) directly connected his social location as an African American/Black man living in government-supported housing for people with HIV to his negative experiences in medical systems:

*Because [in] the emergency room you get Residents that come [in], so it’s like you’re a guinea pig. And it’s frustrating, especially when you know what’s going on with your body, and you’ve been sick, you know. You’ve been positive or have full-blown AIDS for a certain amount of time. And most people know their bodies. So when they go in, they know what to tell the doctor. But if you’re telling somebody new, they’re going to try something else. And like I said, and if you come from [where] I come from in [my neighborhood], they’re going to give you a hard time. The first thing they want you do is take a [drug test] which a lot of people don’t know. They have to tell you that that’s what they’re doing, and it has to be a reason why they’re doing it. So it’s like I said, you have to learn how to advocate for yourself.*

Marcus’s experiences led him to anticipate discrimination and paternalism in health care settings and to understand his provider’s suspicions that he was using illicit substances as an additional factor in denying him his autonomy. While Marcus’ narrative depicts in large part an Emergency Department following standard protocols to provide appropriate diagnosis and treatment planning, it also highlights the widely divergent lenses by which many AABH-PLWH and providers view these interactions, and the mismatch, in many cases, between patients’ needs and the institutional structures where they seek assistance.

Similar to Marcus’ experience, Leon, also described above, felt he is treated as a “pushover” because of his race:

*I feel some places just treat people like pushovers …just cause I’m Black. It’s like, ‘you’re Black,’ you know what I’m saying?… it’s like, [I’m] just treated a certain way all the time. I just get to the point where it’s like I don’t have a voice. I have to do this because they say so… I don’t own myself. Just occupying this, you know what I’m saying? Just using time or something for somebody else’s purpose.*

Subsequently, Leon decided that his best option was to completely disengage from medical care, in order to preserve his autonomy and dignity. Lawrence (described above) also reported feeling pressured by providers to initiate ART since his diagnosis. Indeed, participants described how health care providers rightly promote ART to their patients as their best chance to live longer and healthier lives, and to virtually eliminate the risk of transmitting HIV to others. However, as Lawrence noted:

*All my doctors would beg me to take the medication. They wanted me to do DOT [Directly Observed Therapy], you know, come in, pick up the medication, and I ain't like that at all… I didn't like being told. I wanted my medicine when I wanted to take it, and you know. I want to do what I wanted to do.*

Isaac was a 63-year-old African American man who had initiated ART, but who withheld his decision to then discontinue ART from his provider in order to “*avoid conflict*” with him, which Isaac feared would negatively affect his ability to receive treatment for his other medical concerns. For Isaac, it was necessary to cede a certain degree of autonomy in order to receive necessary medical care:

*Because I didn’t even want to have the conversation of them trying to convince me to do something that I didn’t want to do anyway. Because he was a relatively nice man and I didn’t want to have a conflict with him, because I had to use him for other medical issues as well. So I didn’t want to alienate the process of going to the doctor for my whole body. I might need him to give me some pills for anxiety or for my whatever I am having, my iron pills or may go to in for a colonoscopy and I didn’t want to have the conflict.*

Marcus, Leon, Lawrence, and Isaac all described mechanisms through which their abilities to make autonomous decisions and indeed, control their bodies, was threatened as a result of their identities as low-income, African American/Black men. In the context of structural racism, and health care settings located in a context of structural racism, perceived pressure to initiate ART resonated strongly with participants’ collective knowledge of the history of medical maltreatment of people of color. As a result, patients reported viewing themselves as potential experimental subjects, and medical treatment was treated with suspicion and associated with non-consensual procedures. In this way, medical distrust and the need for autonomy were closely linked.

#### Over-emphasis on HIV/ART

Within brief health care encounters, participants noted that ART was often prioritized over other concerns. As a result, medical care was characterized as typically lacking a focus on overall quality of life, and attending only minimally to participant’s co-occurring health and social issues. Gerard, a 53-year-old African American man, described feeling as though his provider was either unaware or uninterested in why he had waited so long to initiate ART, which served as a disincentive to initiate HIV medication for him:

*And then that’s what [the doctor] just kept…drilling on that same issue. I’m like, does this guy know anything? … He needs to see the bigger picture here. If you want to help someone, like I said, you got to see the whole picture of what’s going on with this person and the reason why they have waited this long [to take ART]. There has got to be some other things that are going on that you’re not asking these questions. You’re focusing on one thing. It’s good but it’s bad at the same time because it’s not really motivating that person to take their medicine or to start taking medicine. I would walk out of here just as empty as I walked in here. I’m sorry.*

For many participants, one piece of the “*bigger picture*” that providers often missed were participants’ co-occurring medical issues, which were often more burdensome to the patient than HIV. Sam, a 40-year-old Hispanic man, for example, avoided medical treatment for a staphylococcus infection. When he did eventually seek treatment, he experienced providers as ignoring the infection that brought him to the hospital, focusing instead on his decision not to take HIV medication, as he explained:

*I had not dealt with [the staph infection] before, and I was trying to cure it at home, you know. Doing my own medical cures, you know. Cause I didn’t wanna really go to the doctors and expose all my records and stuff. But it got worse and worse and worse. So I finally went. [But] they weren’t talking about the thing that I came there for. They were more focused on, oh, you’ve had HIV all along, and you don’t take medication, and you should be on pills, and I was like, …I don’t wanna see you anymore. I need somebody at the top. I’ve been dealing with (HIV) for longer than you’ve known me these past 20 hours, and whatever the occasion may be, I know what’s going on here. I need help with (the staph infection). Any questions about HIV, you need to ask me, I have the answers for you*.

While we acknowledge that health care providers may have correctly identified a relationship between Sam’s untreated HIV and the staphylococcus infection, a lack of attention to what he considered his most pressing medical needs served as a source of frustration, and an impediment to his receiving care in a timely fashion, and further, did not appear to move him closer to a decision to initiate ART.

Rose, a 50-year-old African American/Black woman who viewed her own concerns as being subordinated to the imperative of ART initiation, expressed similar concerns to those of Gerard and Sam:

*Everybody just see the pill. Y’know what I mean? But I’m bigger than the pill. Y’know, the pill is within me, I guess [chuckles]. But the pill is a big thing, y’know what I mean, I don’t know, there’s some kind of way that human got it backwards in their minds, they see the pill as bigger than me.*

Notably, throughout the study we found women in particular were likely to note that while the negative effects of HIV stigma on social relationships were asymmetrically felt by women, and while the disproportionate burden of childcare and other familial responsibilities fell to women, health care providers were often inattentive to these concerns, thus exacerbating what was already perceived to be a paternalistic relationship between patient and provider.

Thus through describing experiences of “*feeling like a number*,” “*a guinea pig*,” “*a pushover*,” or as smaller than “*the pill*,” participants routinely highlighted the ways that structural racism and its influence on medical settings, along with an overall lack of concern for the ways that social class and gender complicate concerns related to HIV care, appeared to contribute to an understanding among AABH-PLWH of their being seen as a problem in need of a solution, rather than as a “*whole person*” receiving high-quality medical care.

## Discussion

A substantial proportion of AABH-PLWH in the United States successfully engage in HIV care, take ART with good adherence, and achieve viral suppression – an important public health achievement [4]. The present qualitative study, however, focuses on the critical subpopulation of AABH-PLWH poorly engaged along the HIV care continuum. We used a CRT framework to illuminate specific mechanisms through which structural racism, namely, the macro-level systems that reinforce inequities among racial and ethnic groups [31], play a role in AABH-PLWH’s HIV care and ART decisions and behavior. Drawing on one of the core tenets of CRT, the present study elicits the experiential knowledge of AABH-PLWH to better understand the links among structural racism and sub-optimal engagement along the HIV care continuum. Throughout, we rely on participants’ counter-narratives to highlight how a historical perspective of multiple forms of marginalization circulates in communities in meaningful ways, and shapes understanding of HIV and HIV care. In this manner, a qualitative analysis with an intersectional and CRT lens proved especially useful in articulating the deep, complex, and systemic structural underpinnings of psychosocial barriers to ART and HIV care for low-income AABH-PLWH.

On an institutional level, the effects of structural racism reverberate throughout strained and over-burdened health care systems, as is evident in the dehumanizing manner by which patients often feel treated by health care workers and staff in such settings. There is a growing awareness, in fact, of the structural foundations of health care dissatisfaction. Timmermans and Almeling (2009), for example, described depersonalization of health care and the effects of bureaucratic control as “pathologies” of modern medicine [55]. Similarly, Weiser and colleagues [56] documented widespread dissatisfaction among HIV care providers in publically funded institutions, finding that compared with providers in private practice, more of those serving the lowest-income patients planned to leave HIV practice within 5 years [56]. Yet, higher health provider satisfaction leads to better health outcomes among patients [57, 58].

Past research has demonstrated the negative effects of discrimination based on race, class, and gender in healthcare settings, especially as it relates to poor ART adherence [59, 60]. We did not find in the present study, for the most part, AABH-PLWH experienced overt discrimination from healthcare providers or other clinic staff. Rather, the manifestations of structural racism in the health care setting were described more as a subtly connected series of “micro-aggressions,” that is, everyday exchanges taking place, generally below the level of awareness of well-intentioned members of the dominant culture, which nonetheless sent denigrating messages about, or repeated or affirmed stereotypes about minority groups [61]. Moreover, the present study demonstrates that even before individuals encounter healthcare staff, structural racism is often first experienced as anticipated discrimination and expectations of substandard medical care, which in many cases are reified by ‘run-down’ physical conditions and the lack of individualized treatment in the institution. Indeed, a theme of feeling dehumanized, anonymous, and less than a whole person in health care institutions runs through several of the main findings in the present study, with serious implications for the health and well-being of AABH-PLWH. The effects associated with race were exacerbated when they intersected with other social categories such as class and gender to magnify and differentiate the negative experiences that were described as permeating the healthcare industry as a whole, and as having a profound effect on individuals’ decision-making at different points of engagement along the HIV care continuum. Thus, while the healthcare encounters of African American/Black women and African American/Black men are different, as in the case of an asymmetrical relation to childcare, both nonetheless experience dehumanizing effects. Moreover, although within the context of this study we did not find that race/ethnicity was necessarily more important than other factors, including HIV status, we did find it was more noteworthy to participants.

Similar to past studies [13], we found counter-narratives concerning the origins, past and present treatment, and a potential secret cure of HIV continue to circulate and to have powerful effects on AABH-PLWH’s perceptions of the healthcare industry and HIV treatment. That is, medical distrust, defined here as an understanding of the healthcare industry as discriminatory, ill-informed, and/or injurious [62, 63], functioned as an important manifestation of structural racism, and not a simple misperception on the part of AABH-PLWH, as it relates to ART in particular. Notably, the fear of side effects is closely linked with distrust of the medical establishment and serves as a powerful and common barrier to ART initiation among AABW-PLWH. Indeed, we found side effects often serve as a painful and visceral reminder of past abuses of people of color, as they resonate with participants’ knowledge of the sociocultural and historical legacy of HIV and HIV treatment among members of their community.

Yet fear and medical distrust, even when prominent, do not necessarily eliminate willingness to participate in health care or medical research, as Westergaard and colleagues have noted [64]. We found participants are torn between, or perhaps immobilized by, the desire to engage in health care and take ART on the one hand, and strong desires to avoid negative health care settings and encounters on the other. There is a substantial literature on the role of counter-narrative understandings as impediments to HIV prevention among people of color [65], and this body of research calls for addressing current discrimination within the health care system and acknowledging the origins of counter-narratives in the context of historical discrimination. Further, Self-determination Theory [66] has served as a grounding for interventions to address this type of ambivalence. Self-Determination Theory highlights the importance of the core needs of relatedness, autonomy, and competence [67]. Indeed in a study of HIV-infected women, Quinlivan and colleagues [67] found that unmet needs for self-determination were common and related to poor HIV outcomes, and conclude that interventions that address core self-determination needs, including autonomy, may enhance the motivation for self-care among HIV-infected women [67]. Self-Determination Theory is the accepted theoretical underpinning of Motivational Interviewing [68, 69], an approach considered culturally appropriate and highly efficacious for populations of color [70]. Our own intervention research with AABH-PLWH to address health inequity integrates Motivational Interviewing with CRT, and takes the approach of directly uncovering and exploring counter-narratives, medical distrust, and fear in intervention components, while not necessarily seeking to change or influence these understandings, and also acknowledging and fostering autonomy, relatedness, and competence, the core self-determination needs [17, 71, 72]. Similarly, Wagner and Bogart and colleagues [73] have developed a promising culturally appropriate ART adherence intervention for African American PLWH, one which foregrounds counter-narratives [73].

### Limitations

One general limitation of this study is that its purposive sampling method may limit its generalizability to the population of AABH-PLWH as a whole. Yet purposive, rather than a random sampling method, is consistent with the goals of qualitative research, which aims for depth rather than breadth. Further, the sample selected included few younger AABH-PLWH, a gap that future studies on this topic can address. Last, we acknowledge that the primacy of race/ethnicity and social class in the results over other disadvantaged categories may have emerged, in part, from the larger study’s emphasis on this category over others such as gender.

### Implications

The present study yields a number of implications for clinical settings and health care and social service providers. The CRT approach highlights the notion that health care settings are located in a society characterized by structural racism and must adapt to that context. There is growing awareness of the importance of “structural competency” in the health care setting. Metzl and Hansen [74] argue that health care settings and medical education need to change, including moving beyond the goal of “cultural competency” to address how structures produce health inequalities; that is, to achieve “structural competency.” Further, they provide a model of structural competency, comprised of the following core competencies 1) recognizing the structures that shape clinical interactions; 2) developing an extra-clinical language of structure; 3) rearticulating “cultural” formulations in structural terms; 4) observing and imagining structural interventions, and 5) developing structural humility [74]. Further, health care and social service providers have the opportunity to assist AABH-PLWH in achieving optimal HIV outcomes by providing a forum for patients to openly address fears and concerns rooted in racial/ethnic and class-based inequality; recognizing that delayed initiation of ART is a valid option for AABH-PLWH with serious barriers to ART; attending to the need of AABH-PLWH to be seen as a whole person; and providing a space within which individuals feel comfortable discussing distrust of HIV care and ART with peers. Further, culturally appropriate social/behavioral interventions in the clinic and community-based settings have an important role to play in reducing these racial/ethnic disparities [17, 44, 75, 76].

## Conclusions

AABH-PLWH tend to be insufficiently engaged along the HIV care continuum and evidence worse health outcomes than their White peers. There is growing awareness that structural racism, that is, the macro-level systems that create inequities among racial/ethnic groups [26], has pervasive negative effects on African American/Black and Hispanic populations in the United States. Critical race theory and intersectionality proved to be useful frameworks for uncovering and exploring the principal mechanisms by which these structural factors affect individual health behavior and decisions among AABH-PLWH. Study findings have potential implications for interventions in health care settings.

## Abbreviations

AABH-PLWH: African American/Black and Hispanic persons living with HIV
ART: Antiretroviral therapy
AZT: Zidovudine
CRT: Critical race theory
HIV: Human immunodeficiency virus
NYC: New York City
PLWH: Persons living with HIV
